# A review exploring the overarching burden of Zika virus with emphasis on epidemiological case studies from Brazil

**DOI:** 10.1007/s11356-021-15984-y

**Published:** 2021-09-08

**Authors:** Merve Tunali, Alexandro André Radin, Selma Başıbüyük, Anwar Musah, Iuri Valerio Graciano Borges, Orhan Yenigun, Aisha Aldosery, Patty Kostkova, Wellington P. dos Santos, Tiago Massoni, Livia Marcia Mosso Dutra, Giselle Machado Magalhaes Moreno, Clarisse Lins de Lima, Ana Clara Gomes da Silva, Tércio Ambrizzi, Rosmeri Porfirio da Rocha, Kate E. Jones, Luiza C. Campos

**Affiliations:** 1 Institute of Environmental Sciences, Boğaziçi University, Bebek, 34342 Istanbul, Turkey; 2 Public Health Department, Araucaria City Hall, Araucaria, PR 83702-080 Brazil; 3 UCL Centre for Digital Public Health in Emergencies, Institute for Risk and Disaster Reduction, University College London, London, WC1E 6BT, London, UK; 4 Departamento de Ciências Atmosféricas, Instituto de Astronomia, Geofísica e Ciências Atmosféricas, Universidade de São Paulo, São Paulo, SP 05508-090 Brazil; 5 School of Engineering, European University of Lefke, Lefke, North Cyprus, Turkey; 6 Department of Biomedical Engineering, Federal University of Pernambuco, Recife, PE 50740-550 Brazil; 7 Department Systems and Computing, Federal University of Campina Grande, Campina Grande, PB 58429-900 Brazil; 8 Polytechnic School of Pernambuco, University of Pernambuco (Poli-UPE), Recife, PE 50720-001 Brazil; 9 Department of Genetics, Evolution and Environment, Centre for Biodiversity and Environment Research, University College London, WC1E 6BT, London, UK; 10 Department of Civil, Environmental and Geomatic Engineering, University College London, WC1E 6BT, London, UK

**Keywords:** *Ae.* mosquito, Arbovirus, Environmental conditions, Precipitation, Sanitation, Social conditions

## Abstract

This paper explores the main factors for mosquito-borne transmission of the Zika virus by focusing on environmental, anthropogenic, and social risks. A literature review was conducted bringing together related information from this genre of research from peer-reviewed publications. It was observed that environmental conditions, especially precipitation, humidity, and temperature, played a role in the transmission. Furthermore, anthropogenic factors including sanitation, urbanization, and environmental pollution promote the transmission by affecting the mosquito density. In addition, socioeconomic factors such as poverty as well as social inequality and low-quality housing have also an impact since these are social factors that limit access to certain facilities or infrastructure which, in turn, promote transmission when absent (e.g., piped water and screened windows). Finally, the paper presents short-, mid-, and long-term preventative solutions together with future perspectives. This is the first review exploring the effects of anthropogenic aspects on Zika transmission with a special emphasis in Brazil.

## Introduction

Zika virus (ZIKV) is an arthropod-borne virus that belongs to the family Flaviviridae and the genus of Flavivirus (Musso and Gubler 2016). The first human infection cases were reported in Nigeria back in the early 1950s (Plourde and Bloch 2016), and the infections were rarely seen until they emerged in the Pacific and Americas more recently (Musso and Gubler 2016). Outbreaks were seen in countries in America, Singapore, Cape Verde, and Pacific countries. During 2015–2016, the pandemic spread to 48 countries in the Americas and Caribbean (Baud et al. 2017) including French Polynesia, Cook Islands, Easter Island, and New Caledonia (Plourde and Bloch 2016). Brazil is one of the main countries that has suffered from ZIKV-related diseases. It was estimated that ZIKV introduction to Brazil , where 440,000–1,300,000 people have been infected by February 2016 (Plourde and Bloch 2016), first occurred between August 2013 and April 2014 (Zhang et al. 2017).

ZIKV can be transmitted through vectors, as well as other non-vector modes via unprotected sexual contact and blood transfusions (Lowe et al. 2018) and by modes of vertical transmission from a pregnant woman to her fetus (Osorio-de-Castro et al. 2017). The infection may be asymptomatic as well as symptomatic with the latter being commonly accompanied with fever, headache, rash, arthralgia, conjunctivitis, and myalgia (Zhang et al. 2021). ZIKV infection is also associated with major viral diseases including dengue, chikungunya, yellow fever, and neglected tropical diseases. The epidemic had a variety of impacts on public health causing neurological impairments including Guillain-Barre Syndrome and microcephaly for the newborns (Lowe et al. 2018). Vélez and Diniz (2016) indicated that ZIKV is also related to reproductive health since it has an effect on fetal development causing microcephaly. The detection of the virus can be done by many methods including PT-PCR, MAC-ELISA/PRNT recommended by the US Centers for Disease Control and Prevention, as well as serological and molecular assays that have some limitations (Zhang et al. 2021).

ZIKV can be regarded as one of the major emerging mosquito-borne viruses that threaten public health globally (Rather et al. 2017; El-Sayed and Kamel 2020; Lequime et al. 2020). The present study holds a vector-borne perspective of the ZIKV; thus, the focus will be given to transmission via mosquitoes and related conditions. The transmission can be based on a combination of risk factors such as vector population, environmental conditions (Lowe et al. 2018), geographical conditions, climate variations, and vector characteristics (Osorio-de-Castro et al. 2017). Therefore, the aim of this paper is to explore the transmission routes by considering the selected set of risk factors using motivating epidemiological case studies from Brazil, to present preventative solutions, and give insights on future perspectives. To the authors’ knowledge, this is the first review exploring the effects of anthropogenic aspects on Zika transmission with an emphasis in Brazil.

## Methodology

The body of literature included in this review covers the main aspects of the ZIKV transmission in Brazil and especially its notable risk factors (i.e., environmental, anthropogenic, and social) as well as the means for preventing its transmission cycle. Readers should keep in mind that although the review was focused on Brazilian cases, it also presents some global facts. We conducted a thorough search via the following search engines: Google Scholar, Web of Science, and Scielo, and we used the following keywords: “ZIKV,” “ZIKV transmission,” “factors affecting ZIKV,” “sanitation,” “public policies,” and “Brazil.”

## ZIKV transmission

ZIKV can be transmitted through the bite of the vectors including mosquitoes (27 species), ticks (12 species), and unknown vectors (14 species) (Song et al. 2017). Mosquitoes are known as the most crucial carriers of ZIKV. The virus can be transmitted via the saliva of the mosquitoes and infects other hosts (Zhang et al. 2021). Several factors affect the transmission of ZIKV including environmental conditions that define the suitability of the parameters to sustain mosquitoes, as well as anthropogenic and socioeconomic conditions that may promote favorable environmental breeding sites for mosquitoes.

### Breeding sites of *Ae.* mosquitoes

ZIKV is mainly transmitted by the female *Aedes (Ae.)* mosquitoes (Zhang et al. 2017), specifically the *Ae. aegypti* and *Ae. albopictus* species (Lowe et al. 2018). Other major species include the *Ae. Africanus*, *Ae. Luteocephalus*, and *Ae. hensilli* (Plourde and Bloch 2016). The *Aedes* mosquitoes are commonly found in the Global South and are typically native to the warm tropical and subtropical regions (i.e., Latin America, sub-Saharan Africa, and Southeast Asia). The *Ae. aegypti* and *Ae. albopictus* have a broad geographic span, while the distribution of the *Ae. luteocephalus* and *Ae. hensilli* is concentrated in Africa and the Pacific Islands, respectively (Plourde and Bloch 2016).

Both *Ae. aegypti* and *Ae. albopictus* can breed in natural and artificial containers (Lowe et al. 2018; Rubio-Solis et al. 2019) and need standing water or water containing organic matter in order to fulfill their growth cycle (Marcondes and Ximenes 2016). These containers include concrete tanks, coconut shells, discarded tires, flower pots, drums, ant raps, and earthen jars (Simard et al. 2005). Larvae and pupae were found most in tires by 33.33% followed by plastic drums, jerrycans, and barrels (24.19%, 19.01%, and 16.04%, respectively) (Getachew et al. 2015). Ferede et al. (2018) indicated the *Aedes* presence in discarded tires by 57.5%, mud pots by 30.0%, followed by mud dishes, ditches, and plastic containers by 21.7%, 21.1%, and 14.8%, respectively. Other sites include flowerpots, discarded sinks, plastic bowls, mud pots, dustbins (Getachew et al. 2015), cement tanks, drums (Abílio et al. 2018), and raw sewage (Chitolina et al. 2016).

Water quality also plays a crucial role in the productivity of the breeding habitats. Stagnated water in storage containers and other atmospheric conditions such as ambient relative humidity and temperature may increase the breeding of *Aedes* mosquitoes (Getachew et al. 2015). Generally, more mosquitoes were able to reside and reproduce in water bodies with poor circulation, higher temperatures, and higher organic content (Ferede et al. 2018). Darriet (2016) indicated that 1.3 g/L plant material (rodent food) was needed for *Ae. aegypti* larvae and 0.61 g/L for *Ae. albopictus* to ensure 50% adult emergence. Dom et al. (2013) stated a water quality index considering chemical oxygen demand (COD), biological oxygen demand (BOD), total suspended solids, ammoniacal nitrogen, pH, and dissolved oxygen. The results showed that polluted water, where the index is higher than 60%, is exceptionally favorable for the breeding of *Aedes sp.* mosquitoes.

### Factors affecting the transmission

#### Environmental conditions

Mosquito density plays a crucial role in the transmission. It is predicted that globally over 2.17 billion live in areas that have environmental conditions that are favorable for ZIKV transmission, including the geographies that have not reported ZIKV cases yet (Messina et al. 2016).

The activity and density of the vectors are highly dependent on environmental conditions including climatic factors (Rees et al. 2018) ), annual cumulative precipitation, temperature suitability, and minimum relative humidity (Messina et al. 2016). Tesla et al. (2018) stated that temperature is a major driver of ZIKV transmission. The study showed ZIKV transmission by *Ae. aegypti* was optimized at 29 °C, with a thermal range of 22.7–34.7 °C. As the temperature increases, the egg and larvae development increases, together with increased fertilization (Filho et al. 2018).

In addition, annual rainfall was found to have an effect on the epidemic. Souza et al. (2018) indicated that the rates of the epidemic were found at highest in the regions where the annual rainfall is highest. The effect of precipitation on population growth of *Ae. aegypti* has mostly been related to local environmental characteristics, especially to the availability and diversity of containers that retain rainwater in the local environment (Santos et al. 2020). Messina et al. (2016) stated that there is evidence that areas with a higher amount of precipitation are generally associated with a higher risk of dengue infection. Several studies showed that the season of rain and monsoon is correlated with the increase of *Aedes* population, providing a good environment for high case rates of ZIKV (Lima et al. 2018; Santos et al. 2020; Thavara et al. 2001). Fuller et al. (2017) indicated that the rainfall-related increase in vector density may have had a significant impact on the outbreak in October 2015. However, they did not find a coupling between temperature or relative humidity and the ZIKV incidences. It is important to note that precipitation seems to be the most important factor compared to temperature when considering environmental conditions in terms of increasing mosquito population as highlighted by Santos et al. (2020). These authors focused on Recife and Fernando de Noronha, two locations positioned near the equatorial line, where temperature does not show a high annual variation, consequently in that case, it would not have an influence on the incidence of *Aedes*. That conclusion, however, does not reject the influence of temperature on the role of increasing *Aedes* population as seen by Lima et al. (2018), where the area of study, placed on Sao Paulo, shows that both temperature and precipitation have a correlation with incidences of the ZIKV vector, *Ae. aegypti*.

Furthermore, humidity is also associated with increased *Ae. aegypti* survival, egg development, and biting rates (da Cruz Ferreira et al. 2017) that promotes the transmission. The selected parameters that may affect the mosquito population with case studies can be seen in Table 1. *Ae. aegypti* populations showed variations based on temporal conditions in Porto Alegre, precipitation rate in Recife, and humidity of Porto Alegre and Recife. Moreover, water quality was shown to affect the mosquito population as shown by the case studies in San Juan Bay estuary and Subang Jaya area in Table 1. On the other hand, natural episodes, such as El Nino Southern Oscillation (ENSO) in Brazil, may promote the transmission since they change the climate conditions, while weather extremes such as droughts increase vector range due to increasing breeding habitats (Filho et al. 2018; Samuel et al. 2018; Vincenti-Gonzalez et al. 2018). The storage of water in urban areas during a drought event can significantly impact the availability of aquatic habitats for mosquito vectors (Brown et al., 2014). For instance, 4 years of drought were seen in Northeastern Brazil prior to the microcephaly cases (Almeida et al. 2019). The environmental conditioning factor in health also contributed to the ZIKV outbreak being more intense in the northeast region than in the rest of the country. This region went through periods of drought, and the outbreak of Zika syndrome coincided with a major drought in the region, between the years 2012 and 2016 (Pedrosa et al. 2020). Samuel et al. (2018) demonstrated that the temperature variations resulting from ENSO drought caused a drastic increase in the development of *Ae. albopictus*.

**Table 1:** Examples of studies that show the impact of different parameters on *Aedes* sp

Parameter	Location/coordinate	Method surveyed	Results	Reference
Precipitation	Recife, Brazil/08° 03′ 14″ S; 34° 52′ 52″ W	The relation between the meteorological data and the egg abundance collected by sentinel ovitraps was analyzed	Egg abundance was positively correlated with precipitation. Highest number of eggs was observed at monthly precipitation of ~500 mm and ~700 mm for Brasília Teimosa and Dois Irmãos/Sítio dos Pintos, respectively	Santos et al. (2020)
		The number of eggs from the collected traps and meteorological data were analyzed	Per unit increase in the levels of precipitation may increase the probability of detection of *Aedes* mosquito larvae by 12.0% for households	Musah et al. (2019)
Temperature	Porto Alegre, Brazil/30° 01′ 40″ S, 51° 13′ 43″ W	*Ae. aegypti* population was monitored, and the meteorological data were incorporated	Weekly minimum temperatures above 18 °C were strongly correlated with increased mosquito abundance. While positive variations in the minimum temperatures of up to 20 °C were related to the growth of the adult mosquito population, fluctuation in minimum temperatures above 20 °C had no impact on the mosquito population	da Cruz Ferreira et al. (2017)
	Campina Grande, Brazil/07° 13′ 14.92″ S, 035° 55′ 1.32″ W, João Pessoa, Brazil /07° 08′ 11″ S, 034° 51′ 9.33″ W, Patos-Jatobá, Brazil/07° 02′ 40′′ S, 037° 16′ 16′′ W	*Ae. aegypti* populations were collected. The life cycles of the mosquitos were investigated under six constant temperatures (16, 22, 28, 33, 36, and 39 °C)	The number of eggs per female was decreased significantly under the temperatures of 16 and 36° C. No significant differences were found in the duration of larval and pupal stages at any temperature for any population. The length of the full life cycle differed significantly among the populations at temperatures between 16 and 22 °C. Specifically, the Campina Grande population required more days to develop	Marinho et al. (2015)
Water quality/flood related	San Juan Bay estuary, Porto Rico/18° 27′ 7.95″ N 66° 6′ 51.04″ W	N, C content of the water-related sources, and *Ae. Aegypti* was monitored together with the ZIKV presence in the mosquitos	Flood increased the nitrogen concentration of the water-related sources. The nutrient content of larval *Ae. aegypti* was highly correlated to the nutrient content of the container of the mosquitos (especially for isotopes 15N, 13C). ZIKV concentration increased significantly with both adult C and N	Yee et al. (2018)
Water quality	Subang Jaya area, Malaysia	Mosquito samples were collected from the selected sites where the water quality parameters were measured. An index was determined as follows: *WQI* = 0.22 × SI DO + 0.19 × SI BOD + 0.16 × SI COD + 0.15 × SI AN + 0.16 × SI SS + 0.12 × SI pH	Breeding of *Aedes* was observed under WQ1 between ~10 and 50 which corresponded as polluted water (highest larvae productivity was observed (25%): DO: 7.71, COD: 654.27, BOD: 447.76, TSS: 568.00, AN: 1.68, pH 7.43)	Dom et al. (2013)
Humidity	Porto Alegre, Brazil/30° 01′ 40″ S, 51° 13′ 43″ W	*Ae. aegypti* population was monitored, and the meteorological data were analyzed	Mosquito abundance steadily decreased when air humidity was higher than 79%	da Cruz Ferreira et al. (2017)
	Recife, Brazil/08° 03′ 14″ S; 34° 52′ 52″ W	The relation between the meteorological data and the egg abundance collected by sentinel ovitraps was analyzed	The humidity was positively associated with the average number of eggs only in Morro da Conceição/Alto José do Pinho among 3 other neighborhood. The average humidity of Recife (80%) was considered ideal for *Aedes* reproduction	Santos et al. (2020)

In addition, climate change can have significant impacts on health, through the spread of vector-borne diseases with increased precipitation in some regions (Filho et al. 2018). Climate change also favors the proliferation of cyanobacteria in lentic water bodies, such as lakes and dams. Cyanobacteria can produce neurotoxins, like saxitoxins, with implications for human and animal health (Marengo et al. 2018), which is discussed in the “Environmental pollution” section. Besides, climate change and climate change-related environmental problems (i.e., drought) may promote transmission, and the virus can be transmitted to northern regions because of the rise in the global temperature (Tesla et al. 2018). For instance, El-Sayed and Kamel (2020) indicated that *Ae. aegypti* which is endemic in Africa also emerged in the Netherlands but could not survive due to the environmental conditions which are currently not suitable for that species. However, a possible rise in the global temperature may also promote the survival of the mosquitoes in the region, affecting the transmission of ZIKV.

So far, we have provided brief evidence of risk factors affecting mosquito-borne transmission from the *Ae.* species whereby such evidence are drawn from studies conducted from various parts of the Global South (e.g., Sub-Saharan Africa, Latin America, and Southeast Asia) where they remain endemic. From here, we narrow the focus of our narrative to a Brazilian intellectual perspective and draw upon evidence based on Brazilian studies only.

#### Anthropogenic factors

**Fig. 1:**
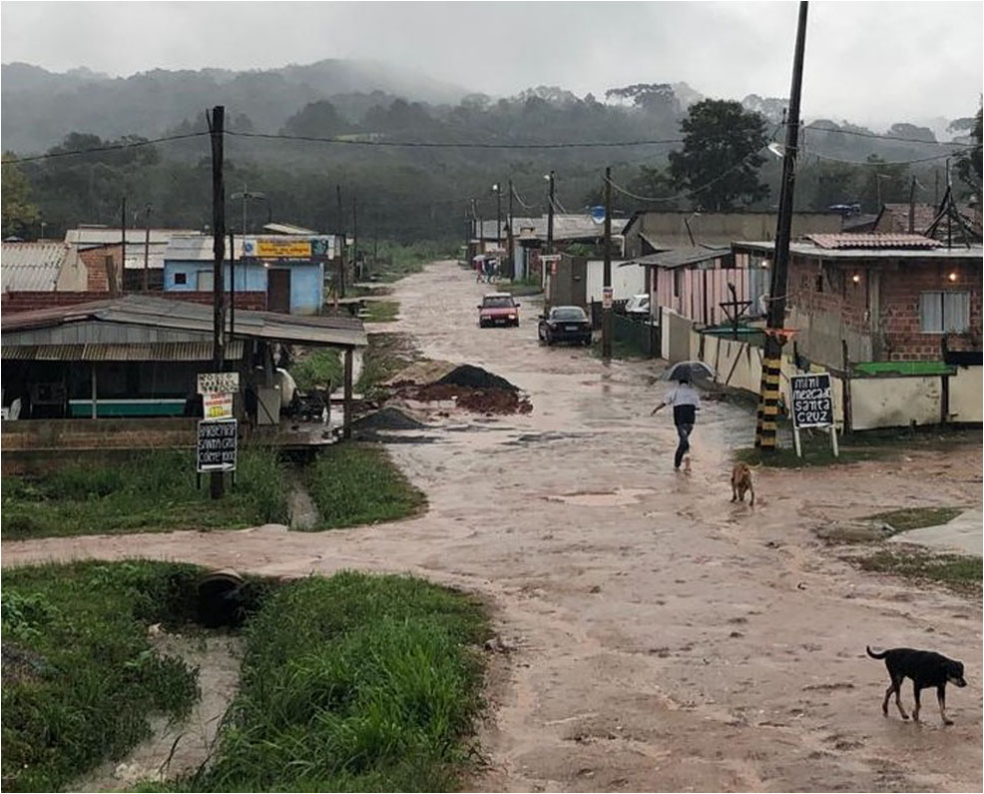
A photograph from Araucária city, Brazil (credit: Alexandro Radin)

Sanitation problems, poor living standards, environmental conditions, and limited infrastructure are among some of the factors that may promote the ZIKV epidemic. Arbovirus epidemics are often the results of areas like the one depicted in Figure 1, just one of the symptoms of the exclusionary development model adopted in Brazil over the centuries, showing total disregard for environmental concerns and resulting in inadequate sanitation, improper disposal of urban waste, and lack of water supply. The incidence of mosquito infestation is highest in densely populated neighborhoods with little vegetation cover and lack of infrastructure, where mosquitoes find hosts more easily (Marcondes and Ximenes 2016). Figure 1 highlights the lack of sanitation due to socioeconomic and environmental conditions, favoring the emergence of the diseases.

##### Sanitation

The relationship between access to sanitation services and the occurrence of arboviruses has been consolidated by research published in Brazil, as in other countries. These studies have identified that deficiencies in the provision of sanitation services are, to some degree and for different contexts, related to the incidence and prevalence of these diseases. Research shows that the occurrence of dengue reflects the social structure of a given territory, its risk, and socio-environmental vulnerability factors. Infestation by the vector is present in several locations, but morbidity is higher in the economically most vulnerable population, with poor sanitation coverage rates (Flauzino et al. 2011; Oliveira and Valla 2001; Vilcarromero et al. 2015; Thammapalo et al. 2008; Carlton et al. 2012; Rey et al. 2010; Braga et al. 2010; Teixeira and Andrade Medronho 2008; Almeida et al. 2009; Lenzi et al. 2000; Costa 2016; Johansen et al. 2018; Pedrosa et al. 2020). It should be noted that the health variables alone do not explain the heterogeneity of the diseases, as their determinants are multiple in aspects and varied factors; however, they are essential elements to explain the occurrence of arboviruses (Santos et al. 2000; Buss and Filho 2007). Therefore, the relationship established between arboviruses in the northeast region, notably ZIKV, and basic sanitation is evident (Sommerfeld and Kroeger 2012; Silva and Machado 2018). For instance, in Teresina (Northeastern Brazil), the main area of microcephaly, has serious problems of sanitation, infrastructure, waste management, and vector control (Almeida et al. 2019).

**Fig. 2:**
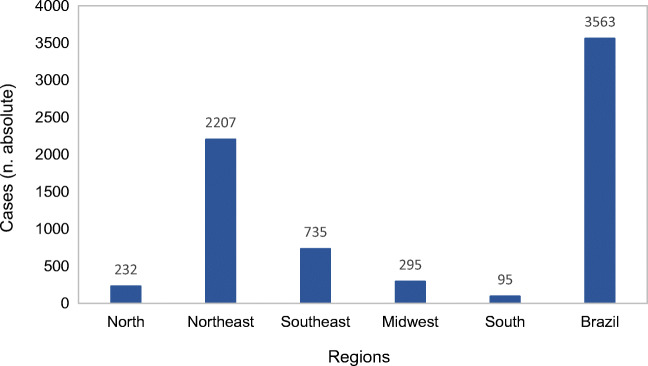
Number of congenital ZIKV cases according to Brazilian regions during the period of 2015 – 2021. (Brasil Ministério da Saúde, 2020)

In October 2015, initially in the State of Pernambuco (Brazil), an unexpected increase in the birth of children with microcephaly associated with the ZIKV was observed months after the confirmation of the native transmission of fever by the ZIKV in Brazil, in April of the same year. For example, microcephaly was observed in 29% of the pregnancies exposed to ZIKV infection in Brazil (Osorio-de-Castro et al. 2017). Almeida et al. (2019) reported 4.46, 6.33, and 3.86 microcephaly incidence rates per 1000 live births for October, November, and December 2015, respectively. Until February 2016, 5,640 suspected cases of microcephaly and 583 cases of this congenital alteration had been confirmed in the country (Brasil Ministério da Saúde (MS) 2016). According to the Epidemiological Bulletin 28 of the Ministry of Health (2020), there are currently 3535 cases of congenital changes caused by the ZIKV since 2015, as can be seen in Figure 2. According to Figure 2, the highest absolute number of cases of congenital changes (microcephaly) caused by ZIKV occurred in the northeast region of Brazil. The 3,563 confirmed cases in Figure 2 were between Epidemiological Week 45/2015 and 25/2020; and consisted of 77.8% (n = 2,749) newborns (less than or equal to 28 days); 15.5% (n = 547) children with an average age of 8.5 months (minimum: 0.0; maximum: 56); and the others (n = 239; 6.8%) corresponded to stillbirths, fetuses, and spontaneous abortions. Sixty-nine fetal deaths were recorded: 13 occurred in 2015, 40 in 2016, five in 2017, six in 2018, one in 2019, and four with an unknown dates. Among live births, 12.4% (409/3,305) died, with a mean age of 10.9 months (minimum: 0.0; maximum: 56). Out of these, nine occurred in 2020 and were residents of Pernambuco (3), Alagoas (2), Bahia (1), Goiás (1), Paraíba, and Santa Catarina (1) (Brasil Ministério da Saúde, 2020). The association between vector proliferation and sanitation is clearly illustrated in Natal, the capital city of the State of Rio Grande do Norte, where a study showed that the majority of Zika cases occurred in the city’s North Zone, where only 5% of sewage is adequately treated (Sousa 2013).

##### Urbanization

**Fig. 3:**
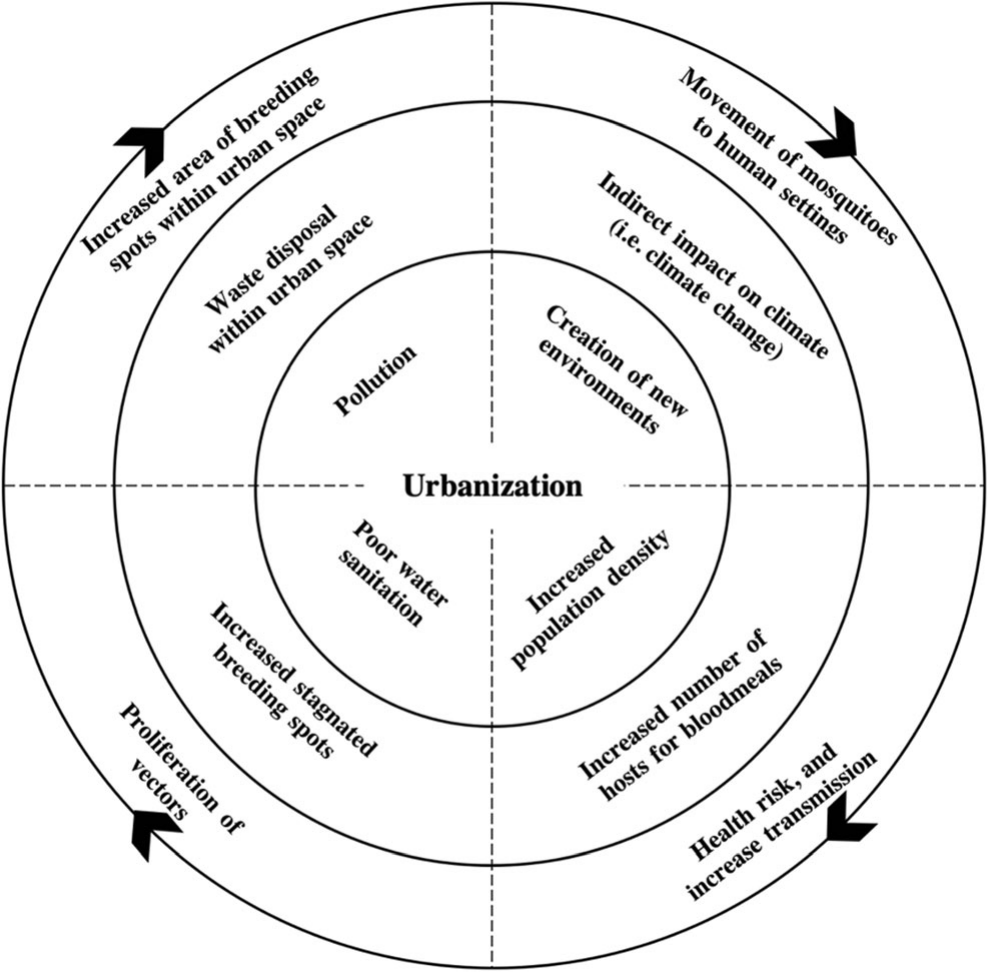
Interaction diagram of unplanned urbanization, illustrating the possible consequences of rapid unplanned urbanization in Brazil

Migration from rural to urban areas and uncontrolled urbanization also affects the transmission since they lead to living in a limited infrastructure (Boecha 2015). It is expected that 70% of the world’s population will be living in cities by 2050, and most of the urban growth will be in low- and middle-income countries (Javed et al. 2017). The urban revolution and growth of cities had and continues to have considerable environmental impacts, particularly in low- and middle-income countries, which do not have effective environmental control legislation. In these countries, rapid and often unplanned urban expansion results in inadequate infrastructure and housing and a lack of essential services such as water supply, sanitation, and waste collection and disposal, posing significant health risks (Gouveia 1999). It is impossible to protect people’s health without taking care of the environment, just as we cannot talk of environmental degradation without mentioning its effects on human health. This discussion involves topics related to soil, water, basic sanitation, diet, housing, and disease, highlighting the close link between urbanization, environmental problems, and health. Fuller et al. (2017) indicated that levels of urbanization may have a significant impact on the ZIKV epidemics in the city of Rio de Janeiro. The interaction diagram shown in Figure 3 schematizes the possible consequences of rapid unplanned urbanization in Brazil (Almeida et al. 2020), highlighting possible impact pathways. The harmony of rapid urbanization accompanied by poor sanitation and lack of infrastructure provides favorable conditions for the *Ae. aegypti* and eventually increases the risk of ZIKV transmission (UNDP, 2017).

##### Environmental pollution

A study published in September 2019 associated, for the first time, the outcome of congenital malformations caused by Zika to the co-factor of contamination by saxitoxins in water for human supply. Saxitoxins are one of the most potent and common neurotoxic substances found in nature (Oliver and Ribeiro 2020). They are water-soluble and run through common water treatment systems (Oliver et al. 2019). Cyanobacteria are well known for their potential to produce cyanotoxins, such as saxitoxins (neurotoxins), which can cause rapid death of animals due to respiratory arrest. The effects on human health can range from intestinal disorders, liver and neuromuscular disorders, allergic reactions, cancer, and death, and a new threat is now added (Oliver et al. 2019). The synergy between saxitoxins and ZIKV, first in vitro, was proven using organoids of the human brain in concentrations of saxitoxins similar to those found in water reservoirs in the northeast region and, later, accompanying pregnant female mice, which consumed water contaminated by saxitoxins and infected by the ZIKV during pregnancy. The authors argue that the northeast of Brazil had a lower number of cases of Zika when compared to other regions, such as the midwest or the southeast but that it had a higher incidence of microcephaly. This was what led them to formulate the hypothesis that cyanobacteria in the water supply would be a causal co-factor for microcephaly associated with ZIKV. These authors also stated that this species is a constant problem. Therefore, this may represent an even more serious threat to public health, since many reservoirs are, or have the potential to be, used in water supply, due to the proximity of populations (Oliver et al. 2019). Furthermore, the causes of cyanobacterial proliferation in urban environments are mainly untreated domestic sewage and soil runoff water. In 2019, only 68.3% of the Brazilian population has access to sewage collection; therefore, more than 100 million Brazilians do not receive this service (IBGE 2020). One must also take into consideration climatic variations, which increase the temperature and intensify the existence of organic matter in the water, conditions that benefit cyanobacteria, according to Oliver et al. (2019).

Associated with the expansion of arboviruses, especially those transmitted by *Ae. aegypti* such as the ZIKV, this factor may represent an increased and more serious health risk for the population supplied with water with the presence of cyanobacteria (Gouveia 1999). In this way, the same climatic conditions that favor the proliferation and dispersion of mosquitoes are those that favor cyanobacterial blooms, with toxins that may be correlated to congenital Zika syndrome. Congenital Zika syndrome may be just the latest warning of the need for a comprehensive sanitation policy for Brazil and other low- and middle-income regions in the world (Camponogara et al. 2013).

### Socioeconomic conditions

Socioeconomic conditions may favor the transmission since it limits access to the services. Vélez and Diniz (2016) indicated that poverty increases transmission due to a lack of piped water, window screens, and insect repellents to limit the mosquito breeding. The Osvaldo Cruz Foundation (Fiocruz), a renowned Brazilian public health research and education institution, researched the profile of families whose babies were born with microcephaly due to the ZIKV in the State of Pernambuco. The results of the research showed that 97% of the babies with microcephaly were born in hospitals in the public health system, 77% of mothers of the babies with microcephaly in Pernambuco are in extreme poverty, families live in precarious situations of handling domestic solid waste, and mainly in places without basic sanitation and where water supply is scarce, and sewage runs out in the environment (Mendonça et al. 2009).

Érico Andrade, professor of philosophy at the Federal University of Pernambuco, conducted an in-depth analysis of causal relations in the spread of the arboviruses where he delimits the boundaries of the “geography of Zika,” dengue, and chikungunya. Andrade affirms that infection reflects urban sprawl, class divisions, and urban segregation, illustrating the close relationship between the ZIKV and microcephaly and social inequality (Brasil Ministério da Saúde 2016). The relationship between social inequality and health is confirmed by Wilkinson and Pickett (2015), who have shown that life expectancy is lower and infant mortality higher in more unequal countries. These authors also suggest that income inequality is such a big problem that improving health in rich countries depends more on reducing inequalities than economic growth (Wilkinson and Pickett 2015).

In Brazil, there are many households that are not connected to public water, and most of the rural houses are not connected to water services (82%), thus storing water for consumption that favors mosquito breeding (The Lancet 2016). The lack of water in the houses makes domestic storage necessary, creating favorable places for the reproduction of the *Ae. aegypti* mosquito (Mújica et al. 2015). In addition, Boechat (2015) indicated that more than 75% of breeding sites arise because of precarious water storage in Brazil. Water storage is necessary for places where there is no clean water supply through a piped system. For storage of water, barrels, plastic drums, and jerrycans are commonly used for domestic use (Getachew et al. 2015), which may favor the breeding. In Brazil, especially in large cities, the elimination of *Ae. aegypti* breeding sites, ZIKV vector, is a complex task, particularly where there are precarious housing conditions, intermittent water supply, inadequate sanitation, and irregular garbage collection,. The mosquito, which lays eggs in domestic water containers and feeds on human blood, favored its spread throughout the world (Costa 2016).

On the other hand, the massive implementation of the selective collection of solid waste, with the separation and appropriate destination for recyclable waste, is an important measure not only for vector control, but also from an environmental perspective. Open sewers, where deposited garbage is also found, are another inexhaustible source of breeding grounds for *Ae. aegypti*.

## The epidemiology of Zika in Brazil associated to social and environmental conditions

The epidemic in Brazil can be characterized by high spatial and temporal heterogeneity (Zhang et al. 2017; Sun et al. 2018) and can vary depending on the mobility of humans and mosquito vectors (Zhang et al. 2017). Sun et al. (2018) indicated that the risk of local transmission is mainly concentrated to specific geographical locations. The local transmission is spatially heterogeneous due to socioeconomic and local climate differences.

For Brazil, the coastal cities in the south and large areas in the north reported the highest number of ZIKV cases, while the central region reflected lower suitability for the ZIKV due to low population densities and smaller mosquito populations (Messina et al. 2016). As for the spatial prevalence in the country, Santos (2018) mentioned that the congenital changes (microcephaly) caused by ZIKV present a heterogeneous pattern but occur mainly in children from poor families in wealthier municipalities and close to large cities or in its periphery. The prevalence of a disease is calculated by the total number of confirmed cases of a disease, divided by the total population of a specific city or location and multiplied by 100,000. Still, the authors describe that there are regions in the country where municipalities have not confirmed cases of microcephaly, that is, 4,650 (83.56% of the total municipalities), 33% are from the southeast region, 26% of the northeast, 25% of the south of the country, and 8% of the north and midwest regions. It is possible to point out the common characteristics among these municipalities, i.e., the lowest average temperature in the four seasons, the largest amount of rain in the summer, the highest average altitude, and the longest distance to the state capital.

The states of Minas Gerais (95%), São Paulo (92%), Pará (92%), Acre (90%), Santa Catarina (95%), Rio Grande do Sul (96%), and Paraná (98%) stand out for presenting more than 90% of their municipalities without confirmation of microcephaly cases. It should be noted that Curitiba was the only state capital inserted in this context. However, the municipalities considered to have low prevalence (0.13 to 5 cases/100 thousand inhabitants) are composed of 507, distributed in 55% in the northeast region, 22% in the southeast region, 10% in the midwest region, 8% in the north, and 5% in the south region. They have the higher averages in the variables of the study by Santos and Neto (2018) if compared to the others: per capita income, average income, unemployment rate, percentage of urbanization, demographic density, basic sanitation, and relative humidity. In addition, they have the second-lowest rates of illiteracy, infant mortality, waste disposal in garbage dumps, temperatures in the four seasons, social inequality, fertility rates, and coverage of primary health care.

The average prevalence (6 to 17.5 cases/100 thousand inhabitants) comprises 314 municipalities located 80% in the northeast region; 6% in the southeast, north, and center-west regions; and 2% in the south region. These regions have the worst percentages of the population in households with garbage collection (89.95%). The climatic variables reflect a higher temperature in the summer and a greater amount of rain in the winter. The municipalities with an average prevalence of microcephaly cases have a population covered by 95.58% with primary health care, the second-highest average coverage among the classifications.

**Fig. 4:**
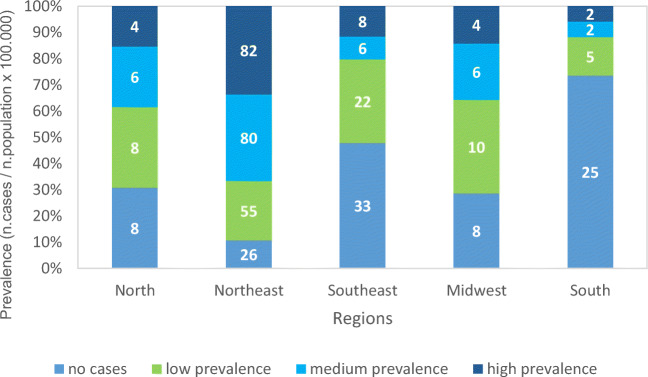
Percentage of Congenital ZIKV Prevalence in the Brazilian Regions during 2016. Data sourced from (Santos (2018)

Finally, the high prevalence (17.5 to 57.26 cases/100 thousand inhabitants) of microcephaly is present in 1.67% of Brazilian municipalities (93), 82% of them located in the northeast, 8% in the southeast, 4% in the midwest and north, and 2% in the south. The averages of the variables in this category suggest infrastructural and socioeconomic vulnerabilities lower per capita incomes, higher concentrations of poor and extremely poor, poorly literacy, with 51.12% of urbanization, and lower demographic density. In addition to low levels of basic sanitation, however, a higher rate of garbage collection, 81.72% carry out the final disposal in illegal and unhealthy dumps, 3.23% in improvised landfills, and 15.05% in regular landfills. Climate indicators point to high temperatures and little rain throughout the year. This information is shown in Figure 4. It can be observed that the high and medium prevalence of cases of congenital alterations (microcephaly) caused by ZIKV was found in the northeast region, a fact that may possibly be related to low conditions of basic sanitation and high temperatures (Santos 2018). This association is also mentioned in a study by the Oswaldo Cruz Foundation (Fiocruz 2017), where the authors report that it is of fundamental importance for the possibility of enhancing the number of cases of arboviruses given the precarious conditions of basic sanitation, contamination of river waters, irregular water supply, and scarce collection of solid waste throughout the country, with emphasis on the northeast region; and they are associated with lower life expectancy and higher mortality, in addition to having negative impacts on infant and maternal mortality (Mújica et al. 2015).

No region in the world is immune to these environmental changes. However, Watts et al. (2015) report that the individual characteristics of each region of Brazil, in addition to the absence of serious public mitigation policies, make the families of children with microcephaly the main martyrs worldwide ZIKV. Since temperature and basic sanitation are undoubtedly significant for influencing the number of cases, the author adds that a special focus is needed on the stimulating factors of global warming and compliance with the laws that govern Brazilian basic sanitation “Therefore*, addressing climate change could be the greatest global health opportunity of the 21*^*st*^ *century*” (Watts et al. 2015).

**Fig. 5:**
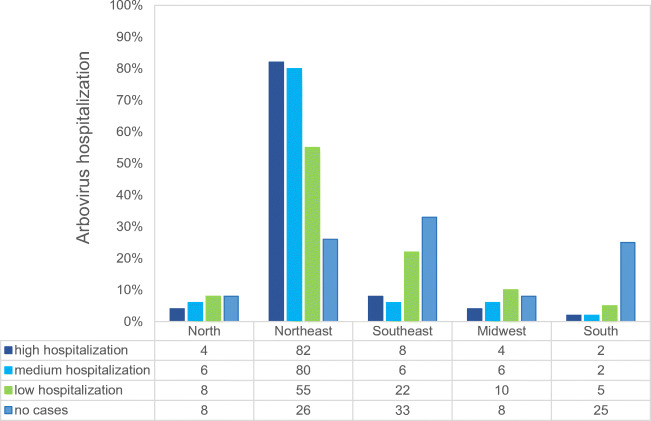
Percentage of Arbovirus Hospitalizations in Brazilian Regions during 2015 and 2021 (Brasil Ministério da Saúde. 2020)

The high incidence rates of arboviruses transmitted by the *Aedes* mosquito in recent years in Brazil (Figure 5) may also be associated with deficiencies in water supply, whether caused by climate change, inadequate management in the provision of services or the lack of public policies, and probably by combining these factors. Even so, they can also be associated with inadequate access to other components of sanitation, such as sanitary sewage, proper management of solid waste, and rainwater drainage (Queiroz et al. 2020).

It can be observed in Figure 5 a peak of hospitalization in 2016, mainly in the northeast region of the country. This peak is associated with the ZIKV epidemic that occurred between the years 2015 and 2016. The hospitalizations for ZIKV reflect the need for specialized and high cost treatment of congenital changes caused by the ZIKV, that is hospital treatment for a long period of life of the patient affected by this pathology.

Therefore, it is of great importance to understand the relationship between access to sanitation services and the occurrence of arboviruses. A vector infestation is present in several locations, but morbidity is higher in the economically most vulnerable population, with poor sanitation coverage rates (Vilani et al. 2014; Vilcarromero et al. 2015; Johansen et al. 2018; Pedrosa et al. 2020). It is important to note that the health variables alone do not explain the heterogeneity of the diseases, because their determinants are multiple in aspects and varied factors; however, they are essential elements to explain the occurrence of arboviruses (Sommerfeld and Kroeger 2012).

## Preventative solutions

### Short- and mid-term solutions

Vector control is the most effective measure that can be taken to prevent ZIKV and other mosquito-borne diseases (Wang et al. 2017; Javed et al. 2017; Lowe et al. 2018; Beltran et al. 2018). An outbreak can be controlled only if the mosquito breeding sites are decreased, and personal precautions are increased in the early stages (Wang et al. 2017). To date, insecticides are mainly used to prevent mosquito-borne diseases; however, these practices are not environmentally sustainable (Han et al. 2016). In addition, insecticide resistance may grow in mosquitoes that results in an unsuccessful application that leads to the change in insecticide type (The Lancet 2016). In addition to these problems, spraying is mainly done in water containers that may have hazardous effects to human and animal health (Osorio-de-Castro et al. 2017).

To combat vector-borne diseases, an integrated approach was recommended by Silva and Machado (2018), consisting of (a) environmental management that eliminates mosquito breeding sites and reducing standing water sites; (b) chemical control where repellents and insecticides may be used; (c) biological control against eggs, larvae, and mosquitoes such as using the seed of *Moringa oleifera* that limit the reproduction of *Ae. aegypti* (Silva and Machado 2018); and (d) release of altered mosquitoes such as Wolbachia-infected male mosquitoes, sterile mosquitoes, and insects carrying a dominant lethal gene (Silva and Machado 2018). Wang et al. (2017) suggested releasing of Wolbachia-harboring female, and male mosquitoes will result in the replacement of *Ae. aegypti* population. Wolbachia bacteria interfere with the reproductive process of the mosquitoes.

Replacement of vector creators is a complex task in Brazilian cities, especially in places where there are precarious conditions in the homes and in their surroundings, poor sanitation systems, and irregular garbage collection (Braga and Valle 2007). However, Benjamin (2016) states that the current campaigns make it appear that the idea is to lead the species to extinction, but this cannot happen. The environment is tropical, and with high temperatures, this scenario favors the reproduction of vector species.

There is an urgent need for major investments aimed at improving the living conditions of the Brazilian population, especially in urban areas. Favorable conditions of access to water and sanitation are fundamental for the prevention of arbovirus. The expansion of the coverage of the selective collection of solid waste, with the separation of the appropriate destination for recyclable waste, is an important measure not only for vector control but also from an environmental perspective (Henriques et al. 2016). Thus, vector control can only be achieved if initiatives in the health sector are accompanied by effective actions in the areas of education, housing, basic sanitation, solid waste, and urbanism (Henriques et al. 2016).

### Long-term solutions

There are no doubt that improving living and sanitation conditions, water supply inadequate levels, supplying piped water, and having sewage systems are some of the suggestions that government and organizations should take for risk reduction (Osorio-de-Castro et al. 2017). The management of the water system is vital as the vectors rely on water reservoirs to complete their biological cycle (Filho et al. 2018), and implementing an integrated water management system will prevent mosquito breeding (Han et al. 2016).

Han et al. (2016) suggested a system that includes a rainwater harvesting that collects, transports, and uses the water where needed, an eco-toilet that separates and stores the feces from the urine composting, and a waterless portable private toilet which is shaped like a box and stores feces and urine separately. Rainwater and eco-toilets can be used as a solution for the challenges in public zones, while an integrated system consisting of a rainwater harvesting system with a soak-away infiltration system and waterless portable private toilet can be used in residential community zones to provide sustainable sanitation options for each family (Han et al. 2016). For a case study, Petelet-Giraud et al. (2018) suggested sustainable water management for Recife Metropolitan region where there are several problems including urban sprawl, unequal distribution of wealth, lack of urban planning, concurrent water uses, lack of supply, and sanitation including regulatory constraints. The water management system can be achieved by (i) strengthening the water resource multi-layer integration to ensure suitable aquifer levels, (ii) strengthening the integration of multi-layered governance to authorize new wells and build partnerships to improve water management, and (iii) raising awareness to preserve water resources.

On the other hand, spreading awareness on the transmittance of vector-borne diseases is one of the crucial measures, since social behavior and individual chores may not be preventative even the community agrees that the water bodies may promote the breeding of mosquitoes (Harris and Carter 2019), and overall development to alter poverty, universal health coverage, enhanced vector control, and developing early warning systems are some of the other preventative measures suggested by Filho et al. (2018). Encouraging low- and middle-income countries to take part in surveillance programs to monitor the diseases is also an important need to stop the epidemics (Javed et al. 2017).

Kostkova et al. (2019) suggested early-warning systems based on prediction via mobile application. The application is able to provide data on the surveillance of the mosquito populations that can be used by the public health authorities for rapid response. The findings of Fuller et al. (2017) suggest an early-warning system based on weather systems may predict the outbreaks before that can help to take precautions.

It is emphasized that a form of organization of the territory will define relations with the environment and, therefore, will not influence the emergence of diseases. With this, it is necessary to observe each territory with its singularities and locate precisely where and how injuries are occurring, what public service the population is in need of, as well as the location of potential health and environmental risk and the areas where vulnerable social situations are concentrated.

## Future perspectives for Brazil

After the decline in the epidemic curve of microcephaly cases in Brazil, the clarification of its causality related to ZIKV infection during pregnancy, and the removal of public health emergencies at national and international levels, important challenges remain for the prevention and the control of ZIKV fever and its consequences in Brazil. These challenges include those related to care for children with microcephaly and their families; reproductive health; gaps in biological, clinical, and epidemiological knowledge regarding ZIKV infection and its complications; and research and development of vaccines and laboratory tests, in addition to the improvement and development of new strategies for the control of *Aedes aegypti* (Garcia 2018).

In addition, due to evidence regarding the sexual transmission of the ZIKV, with the possibility of even asymptomatic cases, reproductive health care becomes more important. The WHO has published a specific recommendation on the prevention of sexual transmission of the ZIKV (WHO 2016), in which it is emphasized that health policies must ensure (i) information on the sexual transmission of the ZIKV; (ii) information on the use of condoms not only as a contraceptive method but also as a barrier to sexually transmitted infections, including HIV and the ZIKV; (iii) counseling on other contraceptive methods for men and women who do not wish to become pregnant; (iv) access to emergency contraception and counseling for women who engaged in sexual activity without a condom; and (v) information for pregnant women and their partners about condom use during sexual intercourse or abstaining from sexual intercourse throughout the entire period of pregnancy are circulated to the entire population for awareness.. Ensuring these actions is still a sensitive and important challenge in Brazil, especially regarding the access of women with a more vulnerable profile to health services.

There are also important challenges in the areas of research and development, in particular for diagnostic tests and vaccines. Existing diagnostic tests are not yet able to accurately diagnose the occurrence of previous ZIKV infection, due to the occurrence of cross-reactions with other flaviviruses. This is a very important limitation in Brazil, where other flaviviruses circulate (mainly dengue and Chikungunya), and a large part of the population is vaccinated against yellow fever. Another challenge is the unavailability of rapid tests (immunochromatographic), serological (IgM and IgG–Elisa), and molecular tests for the timely diagnosis of ZIKV infection, especially in the most vulnerable groups—pregnant women and people with chronic and autoimmune diseases (De Oliveira and Vasconcelos 2016; Duarte and Garcia 2016).

Furthermore, the real consequences of large-scale ZIKV infection still have several open points, as well as the dynamics of cocirculation with other flaviviruses such as Dengue (Valle et al. 2016). There are signs of more severe manifestations of the disease in the occurrence of pre-infection of other arboviruses (Donalisio et al. 2017). Further studies are needed to answer and understand these potential risks, especially considering countries with more than one type of epidemic caused by the viruses of this family. From an entomological point of view, there are also unanswered questions: for example, whether other mosquitoes, in addition to *Ae. aegypti*, are competent to transmit the ZIKV and what conditions are capable of favoring or harming the reproduction of the virus in mosquitoes and hence its vector transmission capability. In this area, several strategies for vector control have been developed and studied (Zara et al. 2016; Yakob and Walker 2016).

As for the determinants and conditions in public health, the serious epidemic of the ZIKV and microcephaly highlights the urgent need for large investments aimed at improving the living conditions of the Brazilian population, especially in urban areas. Favorable conditions for access to water and sanitation are essential for preventing arboviruses. Expanding the coverage of the selective collection of solid waste, with the proper separation and destination for recyclable waste, is an important measure not only for vector control but also from an environmental perspective. The disposal of open sewers, where waste is also found, which constitute another inexhaustible source of breeding sites for *Ae. aegypti*—in addition to other vectors—is necessary. These actions have great potential to reduce the occurrence of diseases and result in longer life expectancy and lower mortality (Henriques et al. 2016).

The Brazilian public health system, with its universal, comprehensive, and equitable character, is responsible for carrying out surveillance and health care actions. Its protagonism was fundamental for the mobilization of all sectors necessary to fight the microcephaly epidemic. However, it lacks the resources to actually fulfill its constitutional attributions and make Brazil overcome the enormous challenge posed by infections caused by the ZIKV and minimize its effects on the Brazilian population.

## Conclusions

Outbreaks of ZIKV that are associated with major viral diseases have been observed in many countries including Brazil. ZIKV is mainly transmitted by *Aedes* mosquitoes, and the transmission is affected by environmental conditions, as well as anthropogenic and socioeconomic factors. The environmental conditions include but are not limited to precipitation, temperature, and humidity as these factors affect the mosquito density which plays a crucial role in the transmission. Anthropogenic factors, especially water and sanitation conditions, urbanization, and environmental pollution, may promote the transmissions since these factors enhance the breeding sites of the mosquitoes. When it comes to socioeconomic factors, poverty may be the reason that increases breeding due to the lack of piped water, window screens, and insect repellents.

In order to combat the epidemics, preventative measures can be taken. Short- and mid-term preventative solutions are vector control that includes but is not limited to environmental management to combat the breeding sites and usage of chemicals to alter the mosquito population. However, vector control should be accompanied by improving living and sanitation conditions and infrastructure in the long term.

## Data Availability

The datasets generated and/or analyzed during the current study are available in the *Ministério da Saúde* repository, https://www.gov.br/saude/pt-br, and *Universidade Federal de Pernambuco* repository, https://www.ufpe.br/ppgecon.
